# A Predictive Model for Selecting Patients with HCV Genotype 3 Chronic Infection with a High Probability of Sustained Virological Response to Peginterferon Alfa-2a/Ribavirin

**DOI:** 10.1371/journal.pone.0150569

**Published:** 2016-03-18

**Authors:** Tarik Asselah, Alex J. Thompson, Robert Flisiak, Manuel Romero-Gomez, Diethelm Messinger, Georgios Bakalos, Mitchell L. Shiffman

**Affiliations:** 1 Centre de Recherche sur l’Inflammation (CRI), UMR 1149 Inserm, Université Paris Diderot, Service d’Hépatologie, AP-HP Hôpital Beaujon, Paris, France; 2 Department of Gastroenterology, St Vincent’s Hospital, University of Melbourne, Melbourne, VIC, Australia; 3 Department of Infectious Diseases and Hepatology, Medical University of Białystok, 15–540, Białystok, Poland; 4 UCM Digestive Diseases and CIBERehd, Valme University Hospital, University of Seville, Seville, Spain; 5 Biostatistics, PROMETRIS GmbH, 68219, Mannheim, Germany; 6 Global Product Development Medical Affairs, F. Hoffmann-La Roche Ltd, 4074, Basel, Switzerland; 7 Liver Institute of Virginia, Bon Secours Health System, Richmond and Newport News, Richmond, VA, United States of America; National Taiwan University Hospital, TAIWAN

## Abstract

**Background:**

Access to direct-acting antiviral agents (DAAs) is restricted in some settings; thus, the European Association for the Study of the Liver recommends dual peginterferon/ribavirin (PegIFN/RBV) therapy wherever DAAs are unavailable. HCV genotype (GT) 3 infection is now the most difficult genotype to eradicate and PegIFN/RBV remains an effective option. The goal of this study was to devise a simple predictive score to identify GT3 patients with a high probability of achieving a sustained virologic response (SVR) with PegIFN alfa-2a/RBV therapy.

**Methods:**

Relationships between baseline characteristics and SVR were explored by multiple logistic regression models and used to develop a simple scoring system to predict SVR using data from 1239 treatment-naive GT3 patients who received PegIFN alfa-2a/RBV for 24 weeks in two large observational cohort studies.

**Results:**

The score was validated using a database of 473 patients. Scores were assigned for six factors as follows: age (years) (≤40: 2 points; >40 but ≤55: 1); bodyweight (kg) (<70: 2; ≥70 but <90: 1); no cirrhosis/transition to cirrhosis (2); ALT ≤2.5 x ULN (1); platelets (10^9^/L) (>200: 2; ≥100 but <200: 1); HCV RNA (<400,000 IU/mL: 1). The points are summed to arrive at a score ranging from 0‒10 where higher scores indicate higher chances of SVR; 141, 123, 203, 249, 232, and 218 patients had total scores of 0‒4, 5, 6, 7, 8, and 9–10, respectively, among whom SVR rates were 45%, 62%, 72%, 76%, 84%, and 89%. Among 622 patients who had scores of 6‒10 and HCV RNA <50 IU/mL by treatment week 4 the SVR rate was 86% (532/622).

**Conclusions:**

A simple baseline scoring system involving age, bodyweight, cirrhosis status, ALT level, platelet count and HCV RNA level can be used to identify treatment-naive Caucasian patients with HCV GT3 infection with a high probability of SVR with PegIFN alfa-2a/RBV therapy.

## Introduction

Hepatitis C virus (HCV) is a major cause of chronic liver disease, with more than 185 million people infected worldwide [1–3]. Globally, HCV genotype (GT) 3 is the second most common genotype, accounting for 22% of all infections [3]. HCV GT3 infection is predominant in South Asia, with a reported prevalence of 54% in India and 79% in Pakistan [3], and there are high rates of prevalence and transmission among intravenous drug users worldwide [4]. Compared with other genotypes, GT3 is associated with a more rapid progression of hepatic fibrosis and steatosis [4;5] and an increased risk of hepatocellular carcinoma [6].

The introduction of all-oral, interferon-free regimens that combine direct-acting antiviral agents (DAAs) has significantly advanced the treatment of chronic hepatitis C [7]. However, improvements in outcomes for GT3 have lagged behind those for other HCV genotypes and the ideal regimen for GT3 remains to be defined [4]. According to guidelines issued by the European Association for the Study of the Liver (EASL), three regimens are recommended for the treatment of patients infected with HCV GT3, namely the combination of sofosbuvir plus peginterferon alfa (PegIFN)/ribavirin (RBV), the interferon-free combination of sofosbuvir plus ribavirin, or the interferon-free combination of sofosbuvir plus daclatasvir [8]. These recommendations reflect the findings of studies such as BOSON [9] in which treatment-naive GT3 patients treated for 12 weeks with sofosbuvir plus PegIFN alfa/RBV had higher sustained virologic response (SVR) rates than patients treated for 24 weeks with sofosbuvir plus RBV (93% vs 84%); the VALENCE study [10], in which an SVR of 85% was achieved in GT3 patients treated for 24 weeks with sofosbuvir plus RBV; and a small study in which an SVR rate of 89% was obtained after 12 weeks of treatment with daclatasvir plus sofosbuvir [11]. The majority of HCV infected patients worldwide have not yet been treated;[12] nonetheless, treatment experienced patients represent an area of high unmet medical need with the EASL guidelines recommending they receive either sofosbuvir plus PegIFN)/RBV or sofosbuvir plus daclatasvir [8]. Consistent with EASL guidelines, those issued by the American Association for the Study of Liver Diseases (AASLD) recommend that treatment-naive GT3 patients without contraindications to interferon receive 12 weeks of treatment with sofosbuvir plus PegIFN/RBV and that those who are not eligible for interferon be treated for 24 weeks with sofosbuvir and RBV [13]. Patients failing prior therapy can receive either sofosbuvir plus RBV for 24 weeks or RBV plus weekly PegIFN for 12 weeks, particularly for patients with cirrhosis [13].

Although DAAs are effective for patients with GT3 infection, access is restricted to patients with advanced fibrosis in many settings [14], an particularly in regions where HCV GT3 has a high prevalence [3], and therefore these DAA-containing regimens may not be readily available to all patients. For this reason, the EASL guidelines continue to recommend dual therapy with PegIFN/RBV in settings where DAAs are not available [8]. PegIFN/RBV is an effective treatment option for patients with HCV GT3 infection [15;16]. In the ACCELERATE trial [15], patients with HCV GT3 had an overall SVR of 66% after 24 weeks of PegIFN alfa-2a/RBV treatment. However, among GT3 patients who achieved a rapid virologic response (RVR), SVR rates were 85%. A retrospective analysis of ACCELERATE [16], restricted to patients who achieved an RVR and received treatment for at least 80% of the planned duration, demonstrated an SVR rate of 90% in GT3 patients treated for 24 weeks. Furthermore, relapse rates were low (7%) in these patients. Therefore, when on-treatment predictors of response such as RVR are used in conjunction with PegIFN/RBV treatment in GT3 patients, SVR rates can be achieved [15;16] that are broadly comparable to those achieved with sofosbuvir-based regimens [10].

The ability to identify which patients are most likely to achieve an SVR with PegIFN alfa-2a/RBV using standard baseline characteristics would be clinically useful in targeting those patients most likely to respond and could be used to avoid delays in treatment. Given the evidence of more rapid disease progression in patients with GT3 infection [4;6], such a strategy would be particularly useful in this large and important subgroup of patients. Alternatively, a patient selection tool could be used to avoid unnecessary treatment and associated adverse events in patients unlikely to respond. Here we present a retrospective analysis of baseline and outcome data from treatment naive-patients with HCV GT3 who received PegIFN alfa-2a/RBV for 24 weeks as part of the PROPHESYS [17] and GUARD-C [18] studies. This analysis was used to develop a simple baseline scoring system which could be easily used in clinical practice to estimate the likelihood of a patient achieving an SVR with PegIFN alfa-2a/RBV.

## Materials and Methods

### Patients and methods

**Fig 1:**
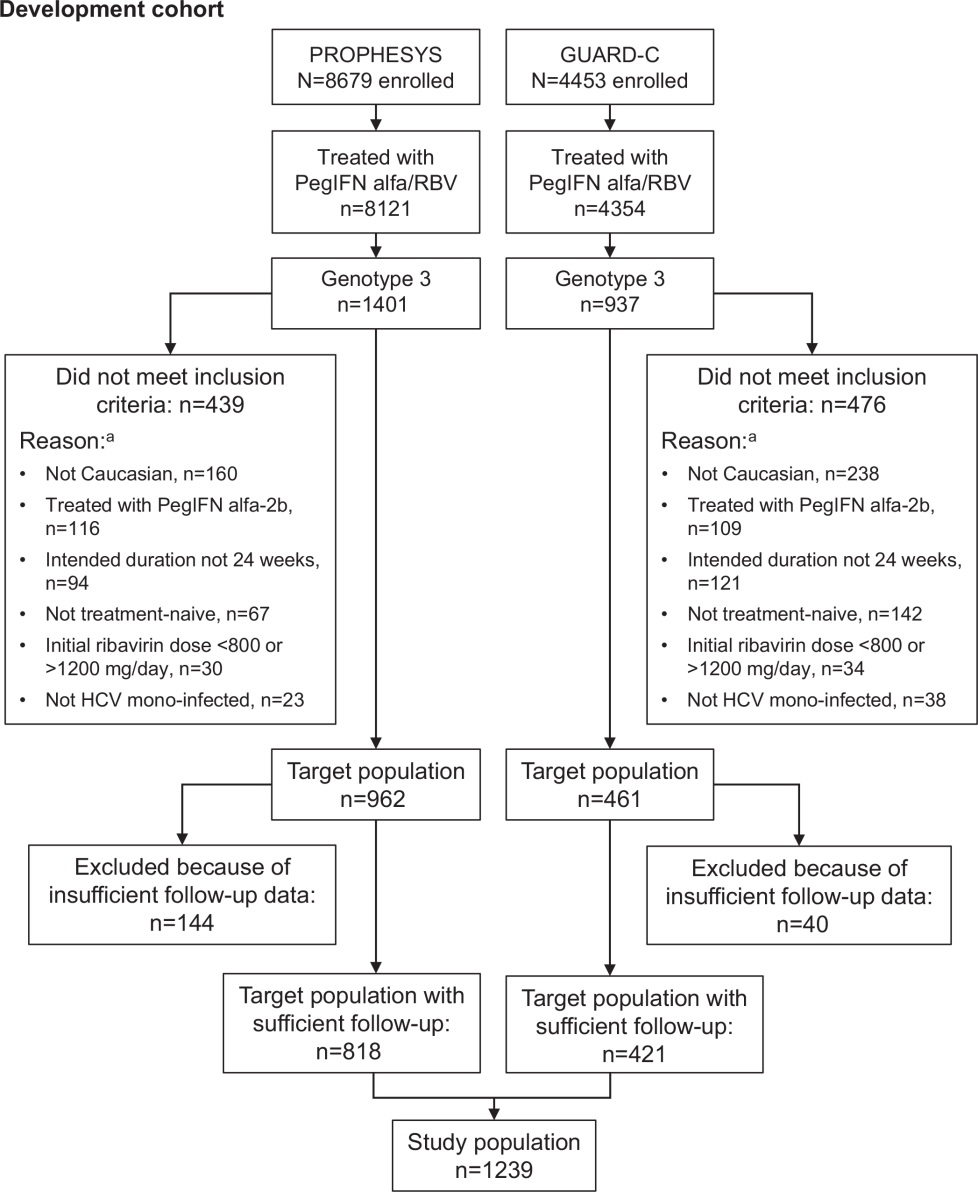
Patient selection for the development cohort.

This was a retrospective analysis of data from 1239 patients with chronic hepatitis C (CHC) and HCV GT3 infection enrolled in the PROPHESYS (ClinicalTrials.gov Identifiers: NCT01070550, NCT01066793 and NCT01066819) [17] and GUARD-C (ClinicalTrials.gov Identifier: NCT01344889) [18] studies (Fig 1). PROPHESYS and GUARD-C were real-world studies conducted in 19 and 25 countries respectively, across Europe, Asia, North Africa and the Middle East, Canada, the USA, and Central and South America.

Patients included in this analysis were adult (≥18 years), treatment-naive Caucasians with CHC, HCV GT3 mono-infection, with baseline serum HCV RNA levels ≥50 IU/mL. Patients had received PegIFN alfa-2a 180 μg once weekly (PEGASYS®, F. Hoffmann-La Roche, Basel, Switzerland) plus RBV (COPEGUS®, Roche) at a planned dose of 800, 1000, or 1200 mg/day for 24 weeks according to the local standard of care and the product license. Patients with cirrhosis were included in both studies: the extent of hepatic fibrosis (if known) was entered by physicians into an electronic case report form as ‘no cirrhosis’ or ‘cirrhosis or transition to cirrhosis’ in PROPHESYS, and ‘no cirrhosis’, ‘transition to cirrhosis’, or ‘cirrhosis’ in GUARD-C. In this analysis, the term ‘cirrhosis’ refers collectively to cirrhosis or transition to cirrhosis. Major exclusion criteria were contraindications to PegIFN alfa-2a/RBV; previous interferon-based treatment, end-stage renal disease and/or major organ transplants; co-infection with hepatitis B virus and/or human immunodeficiency virus and receipt of both PegIFN alfa-2a and PegIFN alfa-2b.

### Ethics statement

The studies were conducted in accordance with the Declaration of Helsinki and the protocols were approved by the Institutional Review Boards/local independent ethics committees at each center. All patients provided informed written consent. Data analyzed in this manuscript are from previously published studies.

### Post-treatment outcomes

Treatment success (SVR) was defined as HCV RNA <50 IU/mL at 24 weeks post-treatment (SVR24). Patients were excluded from the statistical analysis if they had HCV RNA <50 IU/mL at end of treatment (EOT), had no relapse and were missing an HCV RNA measurements ≥140 days after EOT for reasons not related to efficacy or safety (e.g., lost to follow-up). Patients included in the analysis who had missing HCV RNA measurements ≥140 days after EOT were considered to be nonresponders. RVR was defined as HCV RNA <50 IU/mL by week 4. Relapse was defined as serum HCV RNA ≥50 IU/mL during untreated follow-up in a patient with an EOT virologic response, defined as HCV RNA <50 IU/mL at the actual EOT. Only patients with at least one post-treatment HCV RNA measurement were included in calculations of relapse rates.

### Development of baseline predictor score

A simple baseline predictor score was developed to identify patients most likely to respond to treatment. First, generalised additive models (GAMs) were used to explore the relationship between each of the continuous variables and SVR, and to identify appropriate cutoffs for continuous variables to be used in a logistic regression analysis. The values of each factor were grouped by deciles and the empirical probabilities for response at the midpoints of the deciles were also included in GAM plots. Associations between the categorised baseline factors and SVR were then explored by univariate logistic regression (ULR) analyses. Baseline factors considered in the analyses included: age, gender, body weight, BMI, hepatic fibrosis status (cirrhosis versus no cirrhosis [patients with missing information were included as no cirrhosis]), ALT ratio (ALT divided by the upper limit of the normal range [ULN] for the local laboratory), platelet count, HCV RNA level, and planned ribavirin dose.

AST was not recorded in GUARD-C, and thus not considered for the prediction score. All baseline factors with a Wald chi-square p-value of <0.25 were pre-selected for inclusion in multiple logistic regression (MLR) analyses using a backward elimination process to identify independent predictors for SVR. A Wald chi-square p-value <0.1 was required in the selection procedure to retain a baseline variable in the final model. Points (0, 1, or 2) were then assigned for each baseline characteristic in the MLR model based on the magnitude of the parameter estimates in the final model in agreement with the methods proposed by Sullivan et.al. [19] The points were summed for each individual patient, resulting in possible prediction scores ranging from 0 to 10, where higher scores are associated with higher SVR rates. Sensitivity, specificity, positive predictive values (PPV) and negative predictive values (PPV) were calculated for each cut-off of the prediction score.

### Validation of the baseline predictor score

**Fig 2:**
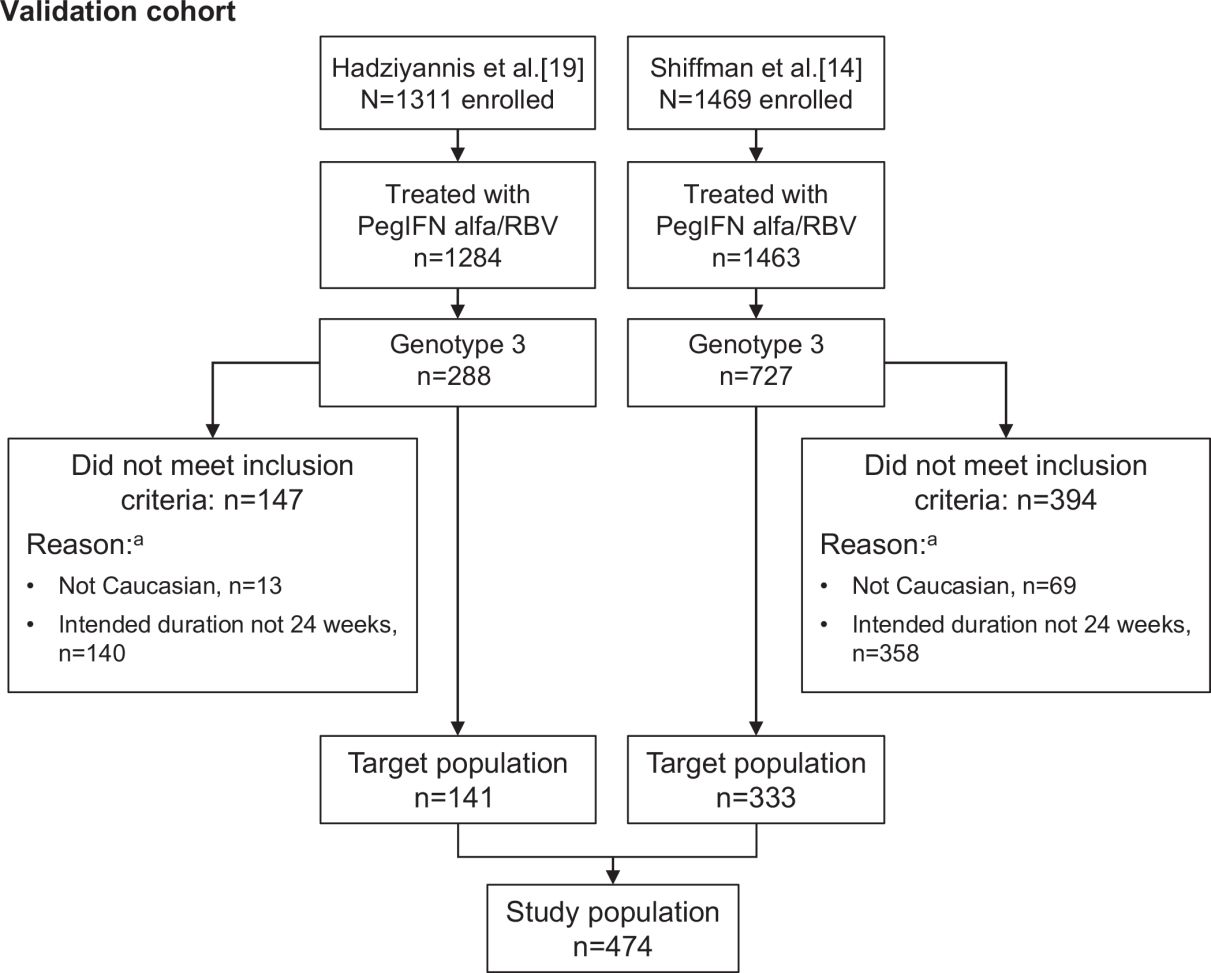
Patient selection for the validation cohort.

The baseline predictor score was validated by comparing the results obtained in the development cohort (i.e., data from PROPHESYS and GUARD-C as described above) with those from patients enrolled in two large randomized clinical trials [15;20]. Only Caucasian patients with GT3 infection who were randomized to 24 weeks of treatment with peginterferon alfa-2a 180 μg/week plus ribavirin at a dose of 800 or 1000/1200 mg/day in the two randomized studies were included in the validation cohort (Fig 2).

## Results

### Patients

Baseline characteristics for the HCV genotype 3 patients included in the analysis are presented in Table 1. The mean age of patients in the model development cohort was 40 years (range: 18–72), 70% were male, and 13% had cirrhosis. The characteristics of patients in the validation cohort were similar to those of the development cohort, although a higher proportions of patients in the former group had transition to cirrhosis/cirrhosis (20.5%) and received an initial ribavirin dose of 800 mg/day (81.9% vs 59.3%). Patients included in the model development cohort came from 26 different countries (**S1 Table**) in Europe, the Middle East, North Africa and North America, with Italy, France and Belgium contributing the largest groups of patients; 271 (21.9%), 185 (14.9%), and 126 (10.2%), respectively. The majority of 474 patients in the validation cohort were from the USA (42.0%), Australia (9.9%), Spain (9.7%) and Germany (9.3%) (**S2 Table**).

**Table 1:** Baseline demographic and disease characteristics.

Characteristic	PegIFN alfa-2a + RBV 24 weeks	
	Model development cohort (N = 1239)	Model validation cohort (N = 474)
**Age, years, mean (±SD)**	40 (10.3)	41 (8.9)
**Gender, n (%)**		
Male	871 (70.3)	314 (66.2)
Female	368 (29.7)	160 (33.8)
**Race, n (%)**		
White/Caucasian	1239 (100.0)	474 (100.0)
**Weight in kg, mean (±SD)**	75.9 (15.5)	78.2 (16.6)
**Body mass index (kg/m**^**2**^**), mean (±SD)**	25.3 (4.5)	26.2 (4.8)
**Cirrhosis status, n (%)**		
Cirrhosis^a^	166 (13.4)	97 (20.5)
No cirrhosis	797 (64.3)	377 (79.5)
Missing	276 (22.3)	-
**ALT ratio, x ULN, mean (±SD)**	2.67 (2.10)	2.89 (1.89)
**Platelets 10**^**9**^**/L, mean (±SD)**	213 (67.1)	227 (59.2)
**HCV RNA, log**_**10**_ **IU/mL, mean (±SD)**	5.78 (0.87)	6.12 (0.80)
**Ribavirin dose, n (%)**		
800 mg	735 (59.3)	386 (81.4)
1000/1200 mg	504 (40.7)	88 (18.6)

ALT = alanine aminotransferase; SD = standard deviation; ULN = upper limit of normal.

^a^ Includes transition to cirrhosis

### Graphic analysis and selection of cutoffs

**Fig 3:**
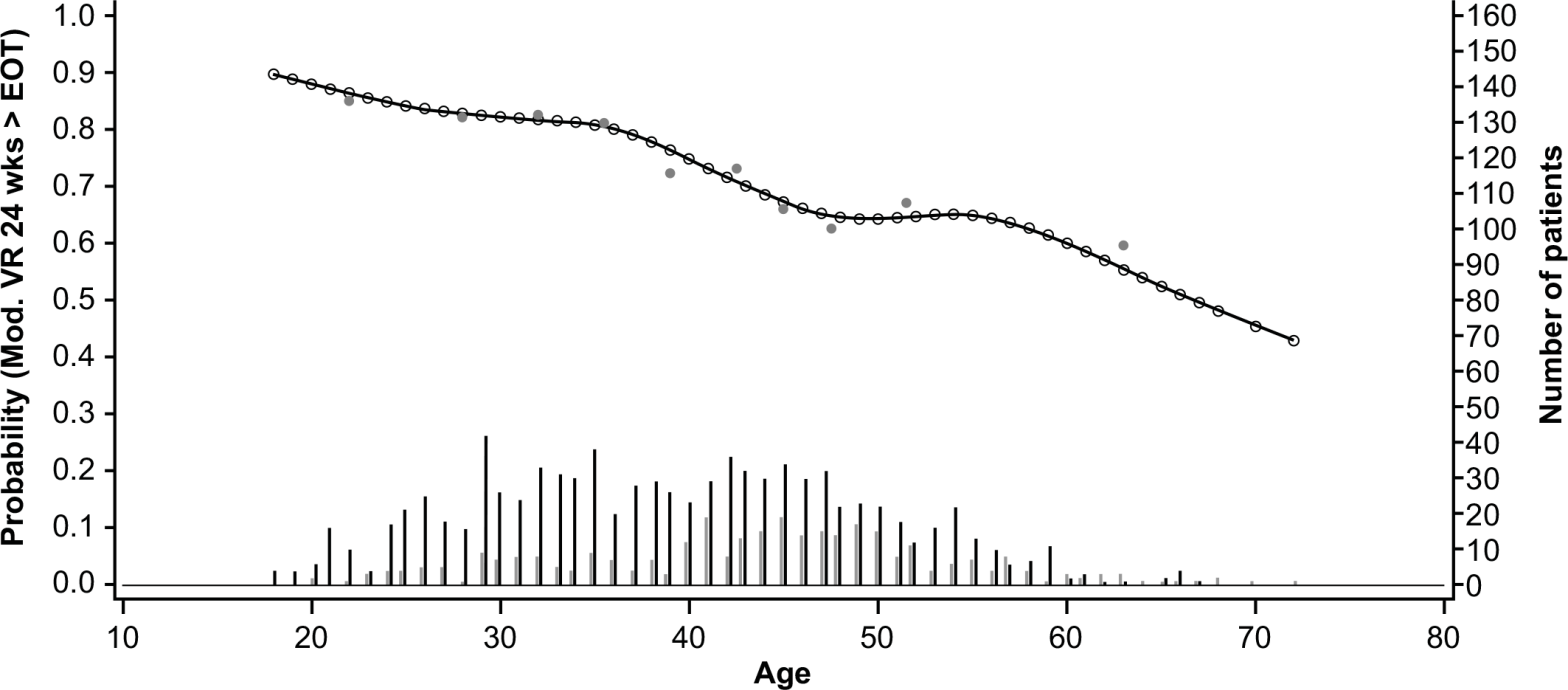
Relationship between age and sustained virologic response (SVR). Short vertical lines indicate the number of patients with SVR (black lines) and no SVR (grey lines). Open circles represent probability of SVR according to the GAM analysis. Closed circles represent empirical probability for midpoints of deciles.

**Fig 4:**
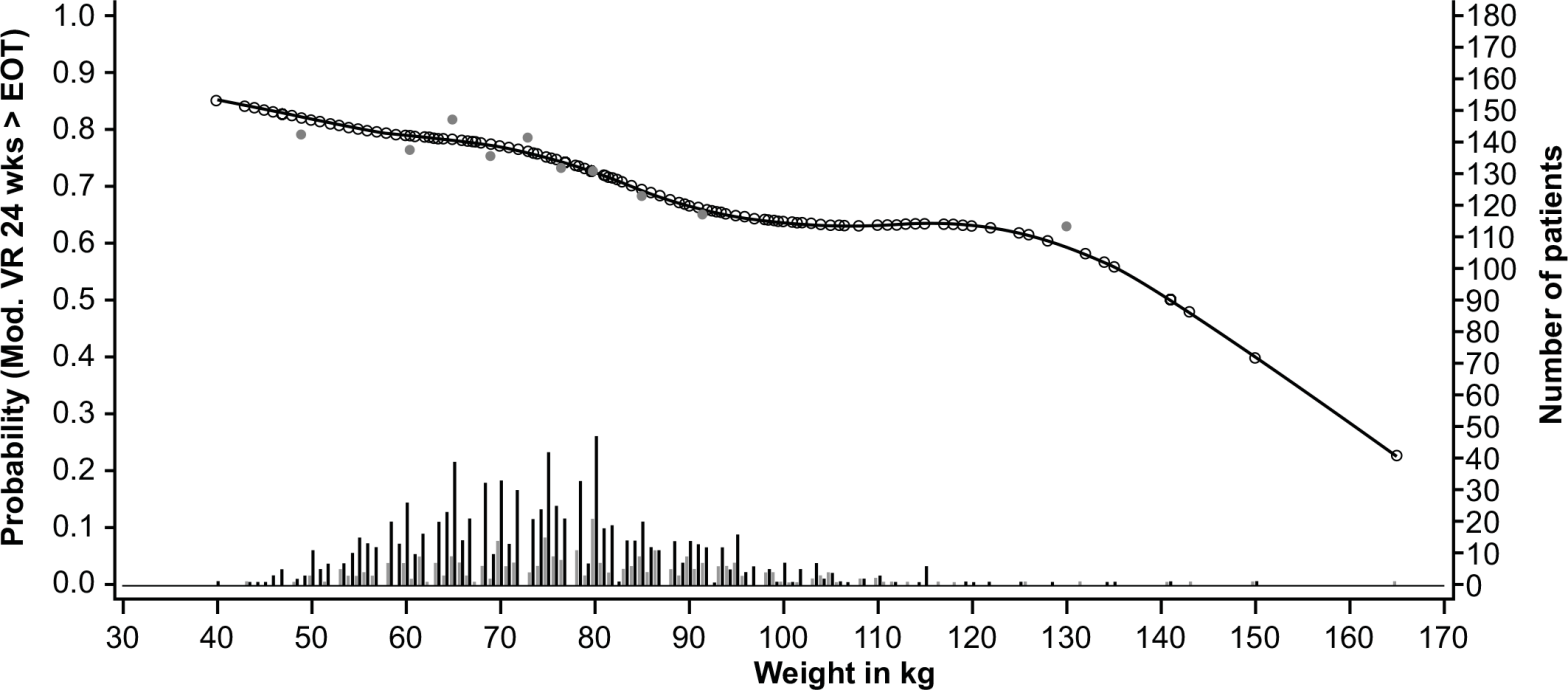
Relationship between bodyweight and sustained virologic response (SVR). Short vertical lines indicate the number of patients with SVR (black lines) and no SVR (grey lines). Open circles represent probability of SVR according to the GAM analysis. Closed circles represent empirical probability for midpoints of deciles.

**Fig 5:**
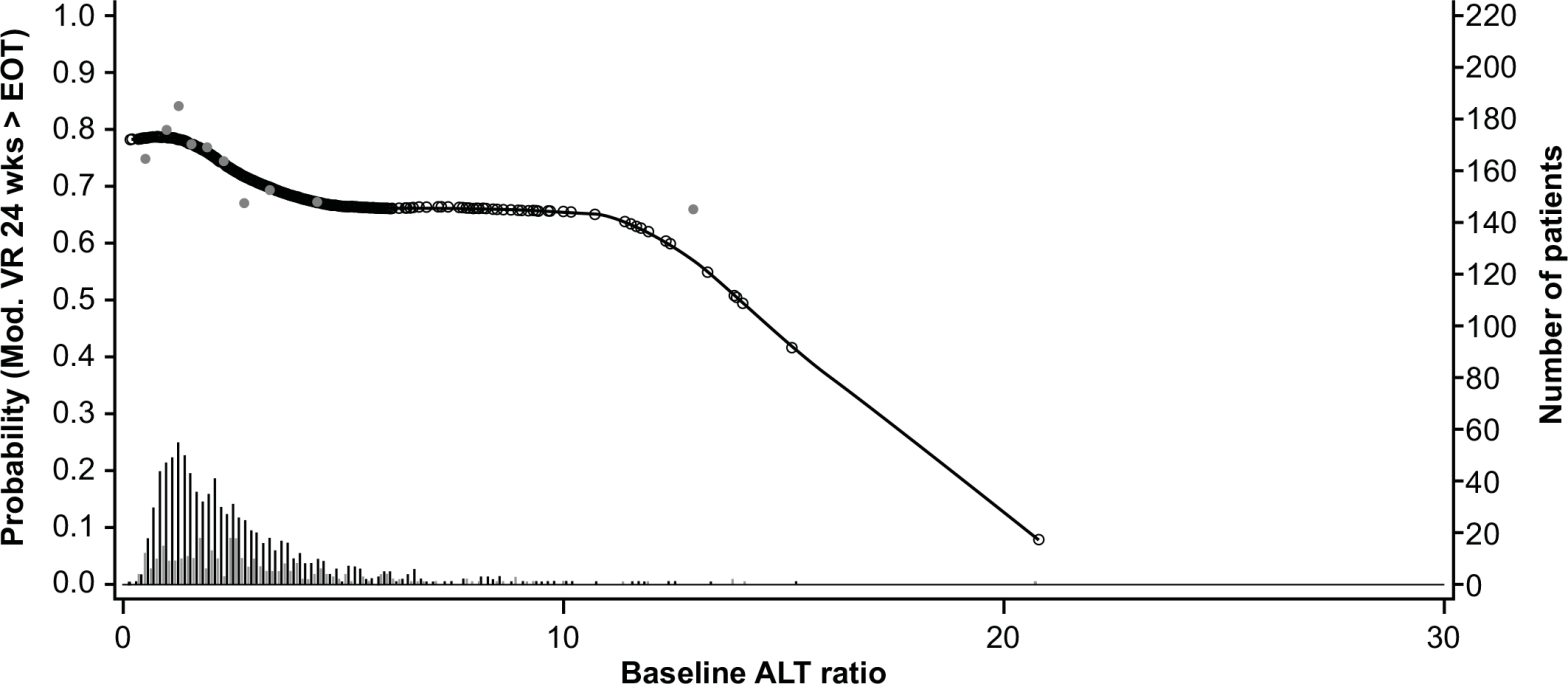
Relationship between alanine aminotransferase (ALT)/upper limit of normal (ULN) and sustained virologic response (SVR). Short vertical lines indicate the number of patients with SVR (black lines) and no SVR (grey lines). Open circles represent probability of SVR according to the GAM analysis. Closed circles represent empirical probability for midpoints of deciles. ALT/ULN = patients' ALT divided by the ULN for the local laboratory

**Fig 6:**
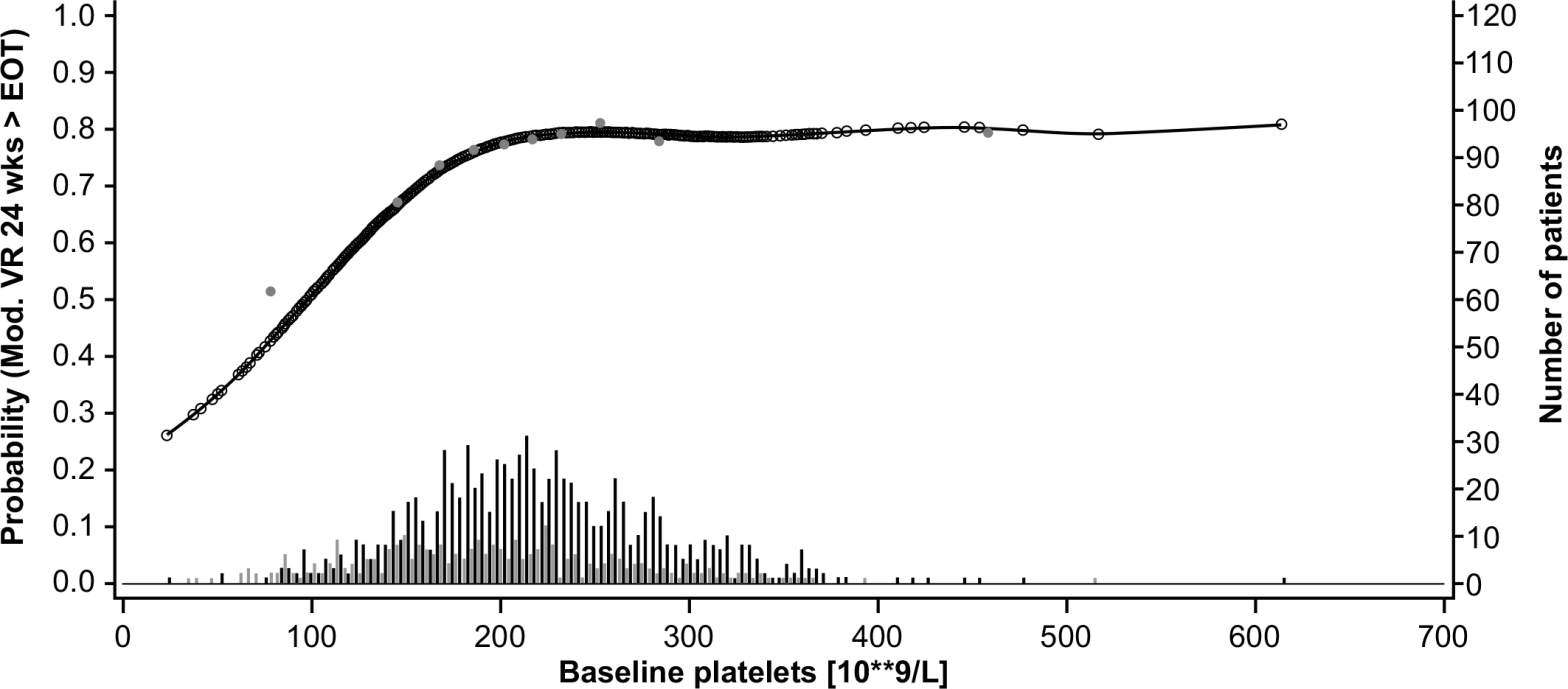
Relationship between platelet count and sustained virologic response (SVR). Short vertical lines indicate the number of patients with SVR (black lines) and no SVR (grey lines). Open circles represent probability of SVR according to the GAM analysis. Closed circles represent empirical probability for midpoints of deciles.

**Fig 7:**
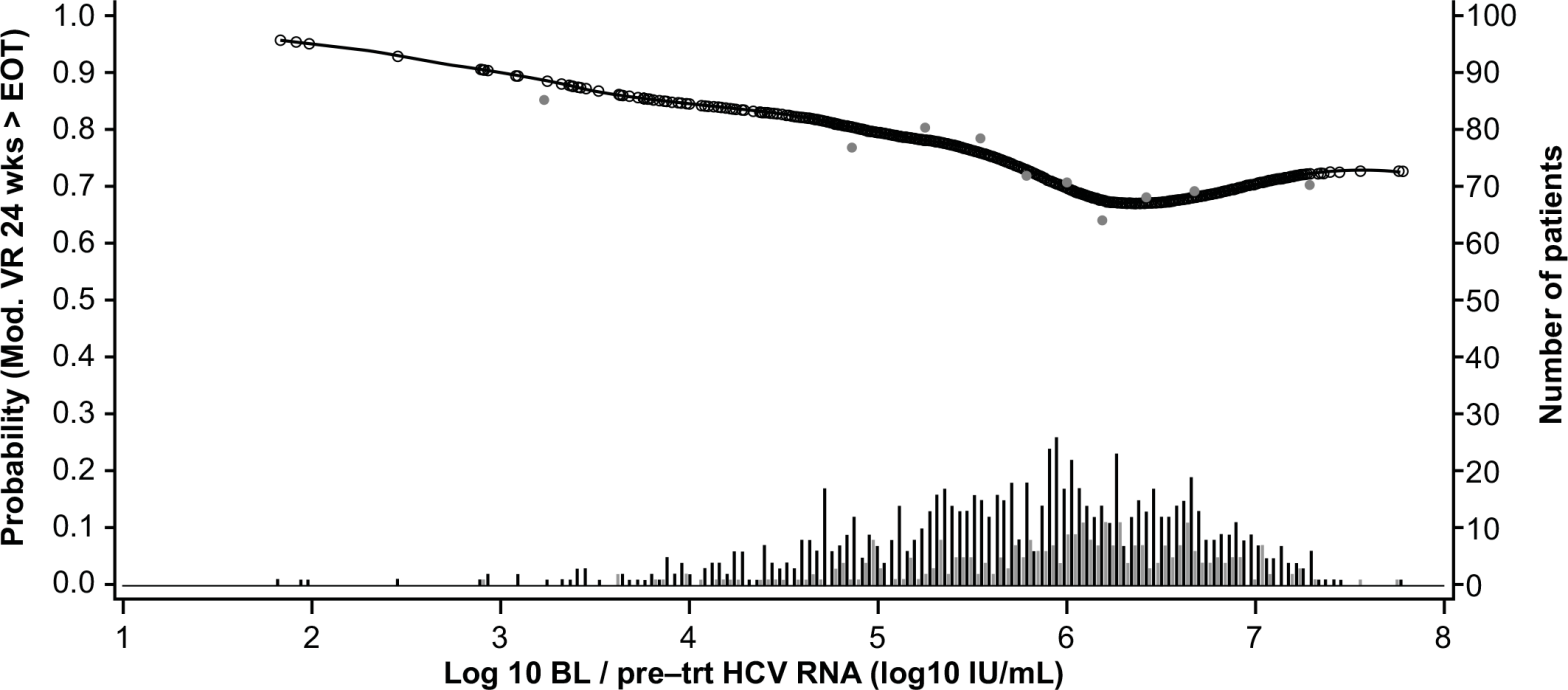
Relationship between serum hepatitis C virus RNA level and sustained virologic response (SVR). Short vertical lines indicate the number of patients with SVR (black lines) and no SVR (grey lines). Open circles represent probability of SVR according to the GAM analysis. Closed circles represent empirical probability for midpoints of deciles.

The GAM plots visualizing the relationship between the independent continuous variables and SVR show a clear increase in SVR rates with lower age, lower bodyweight, lower ALT ratio, higher platelet count, and lower HCV RNA levels (**Figs 3–7**). The relationship between BMI and SVR was similar to that of bodyweight and SVR, but was less predictive. Based on these results, the following cutoffs were used in further analysis; ≤40, 40–≤55, and >55 years for age, <70, 70–90 and ≥90 kg for weight, ≤2.5 and >2.5 for ALT ratio, ≤100, 100–≤200 and >200 x 10^9^/mL for platelets, and <400,000 and ≥400,000 IU/mL for HCV RNA.

### Univariate logistic regression analysis to identify baseline predictive factors

**Fig 8:**
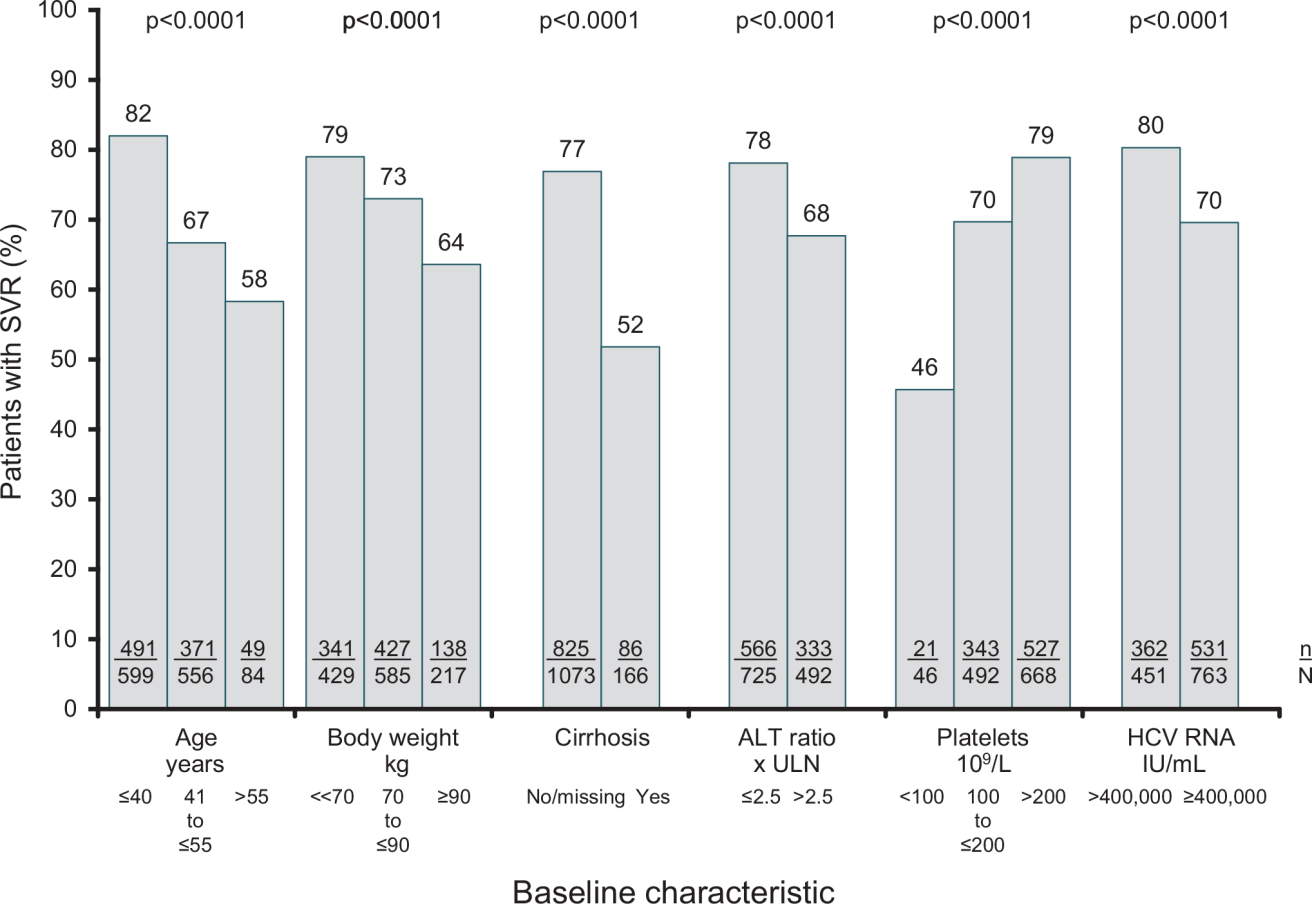
Sustained virologic response by baseline characteristic. ALT = alanine aminotransferase; ALT ratio = ALT divided by ULN for the local laboratory; ULN = upper limit of normal. P-values are based on univariate regression analysis.

ULR analyses revealed that six out of the eight baseline factors examined were predictive of an SVR (Wald chi-square test, p<0.0001, S3 Table): younger age (≤40 years versus 40–≤55 years versus >55 years), lower weight (<70 versus 70–<90 kg versus ≥90 kg), the absence of cirrhosis (no cirrhosis versus cirrhosis), lower ALT ratio (<2.5 versus ≤2.5 x ULN), higher platelet count (>200 versus 100–≤200 versus <100x 10^9^/mL), and lower HCV RNA level (<400,000 versus ≥ 400,000 IU/mL). Rates of SVR in patients by baseline characteristic are shown in Fig 8. Gender (female versus male, p = 0.0805) was only moderately associated with SVR, while planned RBV dose (800 mg/day versus 1000/1200 mg/day, p = 0.8361) was not associated with SVR and was rejected from the model.

### Multiple logistic regression analysis of sustained virologic response

**Fig 9:**
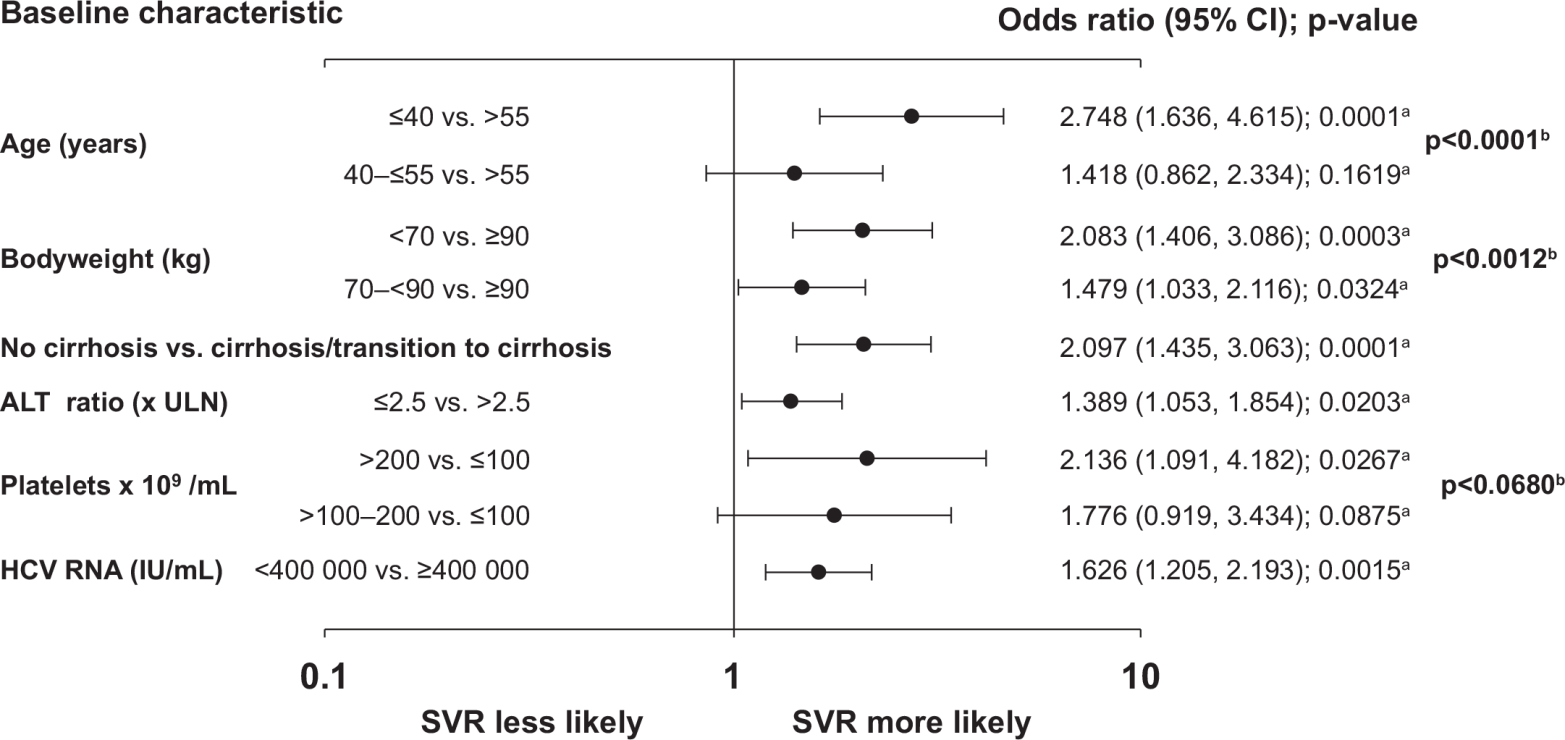
Multivariate logistic regression analysis. ALT = alanine aminotransferase; ALT ratio = ALT divided by ULN for the local laboratory; ULN = upper limit of normal. ^a^Wald chi-square test for each regression coefficient; ^b^ Wald chi-square test for each variable

MLR analysis, using a Wald chi-square p-value of <0.1 in the backward selection process, retained all six factors that were identified by ULR as positive predictors of SVR: younger age (p<0.0001), lower bodyweight (p = 0.0012), absence of cirrhosis (p = 0.0001), lower ALT ratio (p = 0.0203), higher platelet count (p = 0.0680) and lower HCV RNA level (p = 0.0015) (Fig 9 and S3 Table). The formula for estimating the probability of SVR based on the final logistic regression model and three examples is provided in the **Supporting Information**.

The scoring system was developed by assigning a value of 0 points to the reference category for each predictive factor (e.g., age >55 years). Next, points were assigned according to the size of the regression coefficient (S3 Table) using the regression coefficient of 0.4859 for HCV RNA (<400,000 vs ≥400,000 IU/mL) as the unit for 1 point. For example, the regression coefficient for age ≤40 vs > 55 years (1.0108) divided by 0.4859 and rounded to an integer value results in an assigned value of 2 points (**Table 2**). The resulting points assigned for each baseline characteristic ranged from 0 to 2, and the sum of the points assigned to each patient generated a prediction score ranging from 0 to 10.

**Table 2:** Scoring system for predictive baseline characteristics.

Characteristic	Points^b^
**Age, years**	
≤40	2
>40 –≤55	1
>55	0
**Bodyweight, kg**	
<70	2
70–<90	1
≥90	0
**Cirrhosis**^a^	
Yes	0
No	2
**ALT ratio, x ULN:**	
≤2.5	1
>2.5	0
**Baseline platelets, 10**^**9**^**/L:**	
>200	2
100–≤200	1
<100	0
**HCV RNA, IU/mL:**	
<400 000	1
≥400 000	0

ALT = alanine aminotransferase; ALT ratio = ALT divided by ULN for the local laboratory; ULN = upper limit of normal.

^a^ Cirrhosis (Yes) includes patients with cirrhosis or transition to cirrhosis; no cirrhosis (No) includes patients with no cirrhosis and those with missing information.

^b^ The scoring system was developed by assigning 0, 1, or 2 points for each baseline factor that was predictive of a patient achieving an SVR. Points were summed for each patient to give a score ranging from 0 to 10, with higher scores indicating a higher likelihood of SVR.

### Sustained virologic response rates according to baseline prediction score

**Fig 10:**
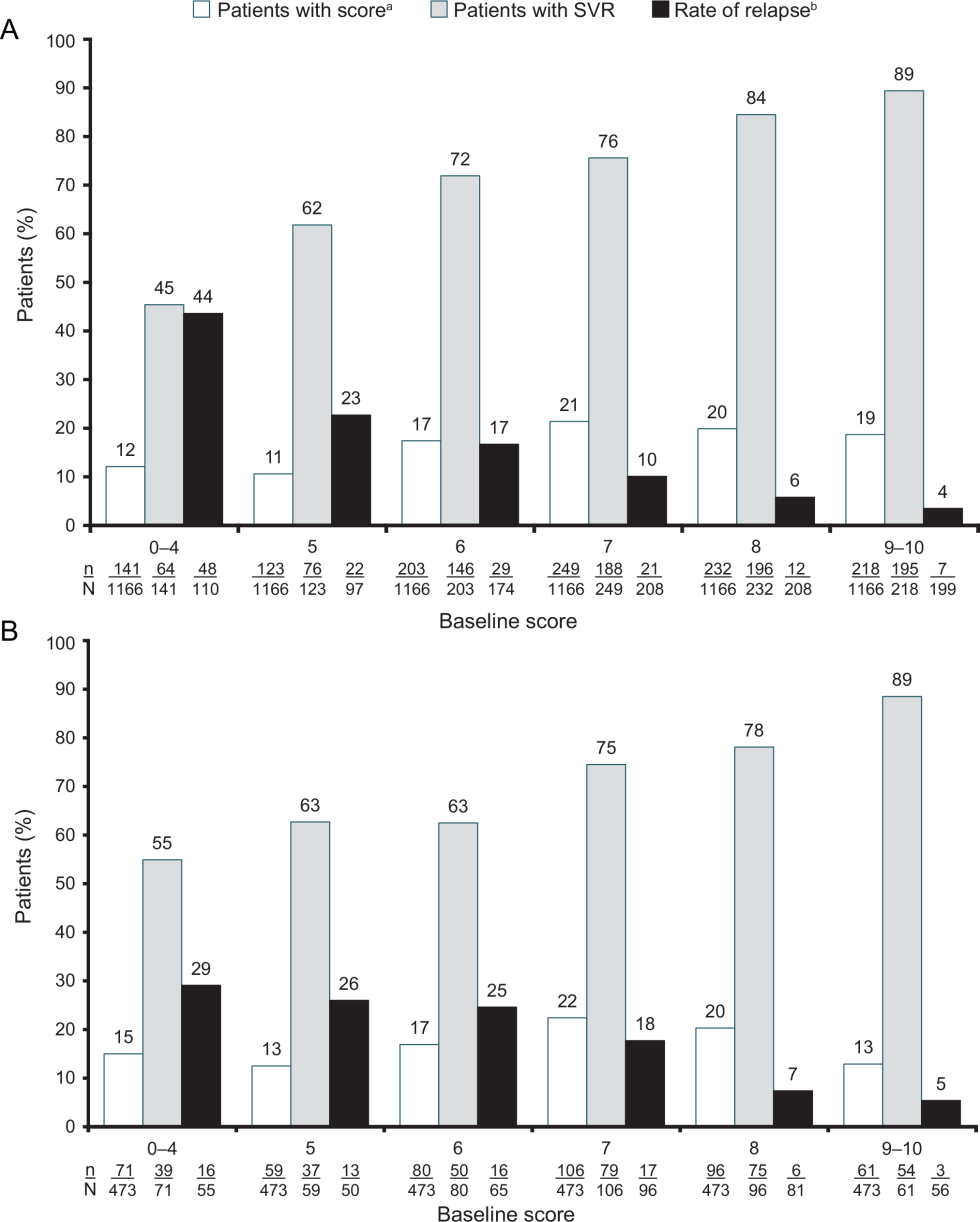
Sustained virologic response and relapse rates in treatment-naive Caucasian patients with HCV genotype 3 by baseline prediction score. A) Development cohort; B) Validation cohort SVR = sustained virologic response 24 weeks post-treatment. ^a^ 73 patients did not have a score and were omitted; of these, 46 (63.0%) achieved SVR. ^b^ Patients with end-of-treatment response and sufficient follow-up.

The distribution of baseline prediction scores in the patient population (N = 1166) was 12.1% (n = 141) with 0–4 points, 10.5% (n = 123) with 5 points, 17.4% (n = 203) with 6 points, 21.4% (n = 249) with 7 points, 19.9% (n = 232) with 8 points and 18.7% (n = 218) with 9–10 points (Fig 10A).

Seventy-three patients did not have a score due to missing values and were analyzed separately. A higher baseline prediction score was associated with a higher rate of SVR, with a score of ≥8 being associated with SVR rates of 87% (391/450; PPV = 87%, see S4 Table), while conversely a score of 0–4 was associated with an SVR rate of 45% (n = 64/141; NPV = 55%, see S4 Table). Higher baseline prediction scores were also associated with progressively lower relapse rates. For example, relapse rates were ≤10% in patients with prediction scores ≥7; in contrast the relapse rate was 44% in patients with a score of 0–4 (Fig 10).

Data from a total of 473 patients treated with the combination of PegIFN alfa-2a plus RBV at a dose of 800 or 1000/1200 mg/day in two clinical trials [15;20] were included in the validation cohort. The distribution of patients with prediction scores in the validation cohort was generally similar to that in the development cohort (Fig 10B) and SVR rates generally increased with the baseline prediction score in a manner that is consistent with the results in the development cohort. For example, the SVR rate in patients with a prediction score of 9‒10 was identical in both cohorts. The trend in relapse rates in the validation cohort was also consistent with that observed in the development cohort. For example, relapse rates in patients with prediction scores of 8 and 9‒10 were 6% and 4% in the development cohort and were 7% and 5% in the validation cohort (Fig 10B).

**Fig 11:**
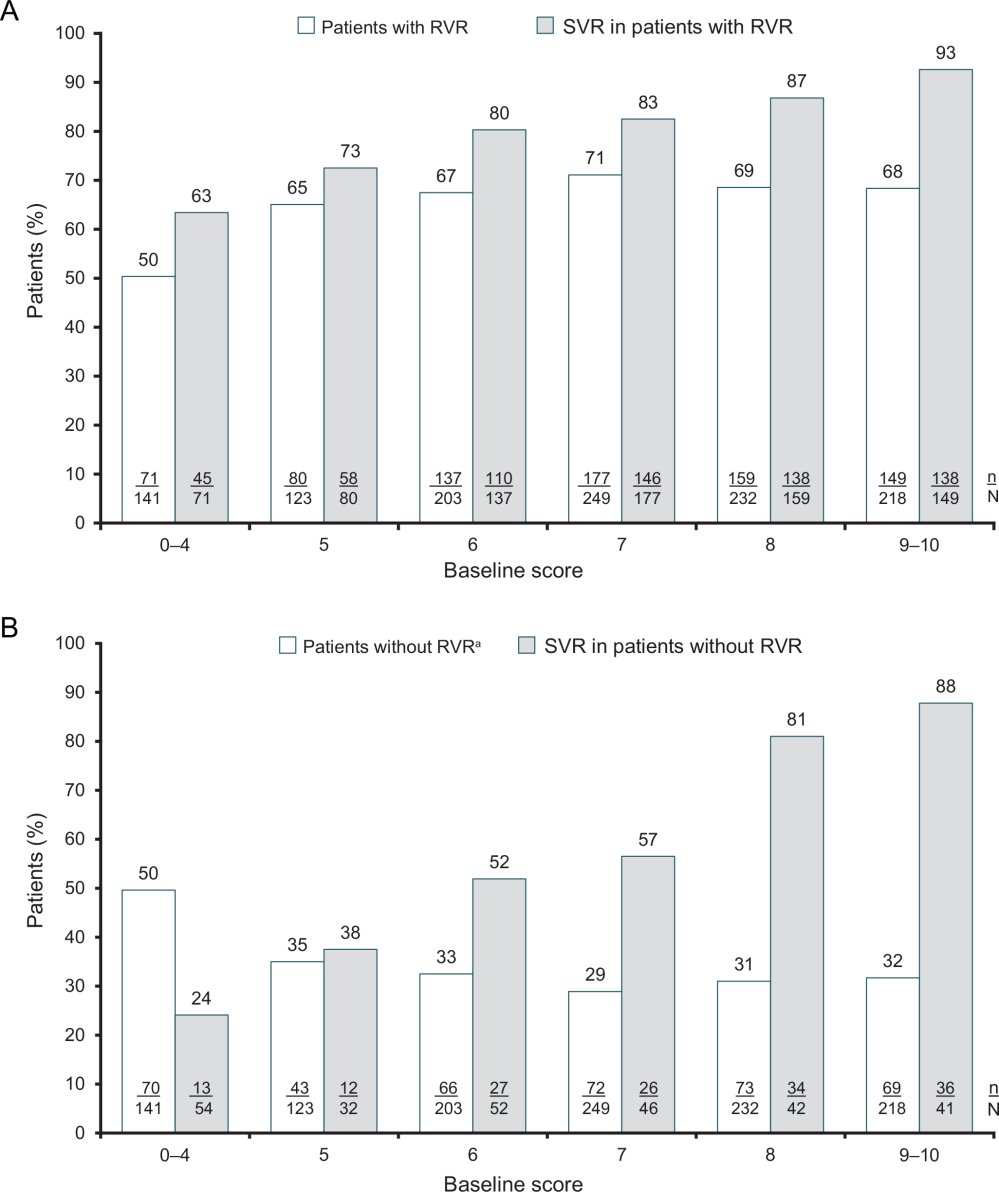
Rapid virologic response (RVR) and sustained virologic response by RVR status in patients with HCV genotype 3 by baseline score. A) Patients with an RVR; B) Patients without an RVR. RVR = rapid virologic response: HCV RNA <50 IU/mL by week 4 of treatment. ^a^ Includes patients with missing Week 4 HCV RNA results.

Among the 1166 patients with a baseline prediction score, 773 (66.3%) individuals achieved an RVR. The overall SVR rate was higher in patients with an RVR (82%, 635/773) than without (55%, 148/267). Baseline score does not appear to have a large influence on achievement of an RVR. For example, 50% of patients with a score of 0‒4 achieved an RVR, and among those patients with higher scores (5‒10), 65% to 71% of patients achieved an RVR (Fig 11A). Among 622 patients who had prediction scores of 6‒10 and achieved an RVR, the SVR rate was 86% (532/622). SVR rates increased consistently and significantly (p<0.0001, Cochran-Armitage trend test) with increasing prediction score regardless of RVR status (Fig 11A and 11B). Among patients with an RVR, SVR rates were lowest among those with a score of 0‒4 (63%) and highest among those with a score of 9‒10 (93%) (Fig 11A). Although SVR rates were consistently lower among patients without an RVR, a similar trend is apparent: the SVR rate was 24% among those patients with a score of 0‒4 and 88% among those with a score of 9‒10 (Fig 11B).

**Fig 12:**
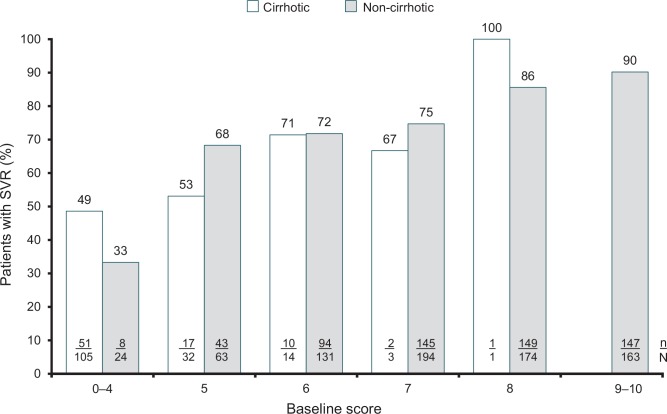
Rate of response in treatment-naive cirrhotic and noncirrhotic Caucasian and patients with HCV genotype 3 by baseline score. Patients with missing scores omitted (noncirrhotic; n = 48, cirrhotic; n = 11). ^a^ Patients with missing information on cirrhosis status were given the same score as no cirrhosis.

As would be expected, the majority of patients with cirrhosis had a low baseline score: 88.4% (137/155) of patients had a score of 0–4 (n = 105) or 5 (n = 32) and a corresponding ~50% chance of achieving an SVR (Fig 12); however, in the limited number of patients with cirrhosis and a higher score (n = 18), an improved response rate was observed; a score of 6 or above corresponded to an SVR ≥71%, which was comparable to noncirrhotic patients.

## Discussion

This analysis demonstrates the possibility of identifying those Caucasian, treatment-naive patients with CHC and HCV GT3 infection who have a high probability of achieving an SVR with PegIFN alfa-2a/RBV treatment, with a simple scoring system that uses readily available and well-known baseline predictors of response (age, bodyweight, cirrhosis status, ALT level, platelet count, and HCV RNA level). Points assigned to each predictive baseline factor were summed to give individual patients scores ranging from 0 to 10. Higher baseline scores were seen to correspond to higher SVR rates such that patients with a score of 6 or above achieved SVR rates of ≥72%, increasing to ≥84% in patients with a score of 8 or above. This study highlights the combined influence of these factors, represented by an easy-to-understand score, which is specific for HCV GT3 patients. Approximately 39% of patients in the development cohort and 33% of patients in the validation cohort had baseline prediction scores of 8‒10, among whom 87% and 82% of patients achieved an SVR, respectively, and approximately 5% and 7% of patients relapsed. The results of the development cohort and validation data sets are thus in close agreement, which is noteworthy because the development cohort was derived from two large and diverse cohort studies (PROPHESYS and GUARD-C) [17;18], while the validation cohort was derived from two large randomized trials with stringent patient selection criteria [15;20].

A similar scoring system, termed the dual therapy response predictor (DTRP) has been developed to select Caucasian, treatment-naive HCV genotype 1 patients with a high probability of achieving an SVR with PegIFN alfa-2a/RBV [21]. Patients were assigned points for age, BMI, ALT and AST levels, platelet count and HCV RNA levels, to give a score between 0–10. Scores were separated into three groups: 0–2, 3–4, and ≥5. Higher scores were associated with higher rates of SVR, with a score of ≥5 leading to SVR rates of 77%.

Recent advances in treatment with newer DAAs have resulted in higher SVR rates with decreased treatment duration [7;8;13]. Nonetheless, dual therapy with PegIFN/RBV remains an important treatment option, even in high-income countries, due to a lack of access to newer DAAs or because of limited healthcare budgets [22]. The relevance of PegIFN/RBV for the treatment of patients infected with HCV genotype 2 or 3 in the DAA era was assessed in Germany. The authors concluded that treatment with PegIFN/RBV for 12–24 weeks was highly effective (SVR = 90%) and comparable to 24 weeks of sofosbuvir plus RBV in well-selected (low baseline viral load, no cirrhosis and an RVR), treatment naive, HCV GT3 patients that adhered to treatment and completed follow-up [22]. However, including patients who did not achieve an RVR, did not complete treatment, or were lost to follow-up in the analysis, resulted in SVR rates of only 48% (intention-to-treat population), highlighting that selection of patients is crucial for achieving high SVR rates with PegIFN/RBV. A number of earlier studies have demonstrated that PegIFN/RBV treatment durations of ≤16 weeks are effective in patients infected with HCV genotype 2 or 3 [16;23–28]. Although these shorter treatment durations are associated with higher relapse rates [29], they would provide economic savings and expose patients to fewer adverse events [27].

RVR is an important on-treatment prediction of response, which is widely used with PegIFN-based therapy. In the ACCELERATE study [15], HCV GT3 patients who achieved an RVR had an SVR rate of 85% compared with 39% for those who did not. Similarly, in the real-world PROPHESYS study [17], HCV GT3 patients who received at least one dose of PegIFN achieved SVR rates of 61% (72% in a sub-analysis of patients who completed treatment and with sufficient follow-up data), which increased to 67% (81% in patients who completed treatment and follow-up) when an RVR was achieved. In this analysis, achieving an RVR increased SVR rates across all score groups; a score of 6 combined with RVR resulted in an SVR rate of 80%, whilst a score of 9–10 and RVR resulted in an SVR rate of 93%. This is comparable to the SVR rates reported for 24 weeks of sofosbuvir and RBV in treatment-naive GT3 patients (94%) [10]. Although patients who did not achieve an RVR had consistently lower SVR rates than patients who did achieve an RVR, SVR rates still increased consistently with baseline prediction score in these patients and exceeded 80% among those with prediction scores of 8 or higher. For this reason, the absence of an RVR should not be used as a strict stopping rule.

Delays in treatment are not advisable for HCV GT3 patients due to their increased risk of fibrosis, steatosis and hepatocellular carcinoma [4;6]. Use of this simple scoring system will aid clinicians in selecting patients who have a high probability of responding well to treatment with PegIFN/RBV, allowing treatment to commence without delay. Patients with a moderate (6–7) or high (≥8) score could begin treatment with PegIFN/RBV and then at week 4 the decision whether to continue treatment could be made on the basis of achieving an RVR, at least in patients with a moderate baseline score. Given that SVR rates exceeded 80% in patients with baseline scores of at least 8 who did not achieve an RVR, it is not recommended that RVR be used as a stopping rule for all patients. Use of the scoring system in combination with RVR would also help minimize exposure to PegIFN and avoid associated adverse effects in patients unlikely to respond to treatment.

The main strengths of this scoring system are, first, the ability to identify GT3 patients with a high probability of achieving an SVR with dual PegIFN alfa-2a/RBV therapy, and, second, the ease of use and ability to calculate the score using routinely available clinical data. Furthermore, using the score in combination with on-treatment prediction of response at 4 weeks (RVR) can be used to optimize treatment and refine patient selection. The scoring system was developed from two large cohort studies [17;18]; there was no relevant difference in SVR rates for each score group between the two studies, allowing the studies to be combined for this analysis. This suggests the scoring system is reliable and applicable to patients in routine clinical practice.

Limitations of this analysis include its retrospective nature; the inclusion of data from Caucasian patients only; the inclusion of data from only treatment naïve patients; the low number of cirrhotic patients; the use of the same score for patients with missing information on cirrhosis and those with no cirrhosis; and the absence of host *IL28B* genotype. The inclusion of only Caucasian patients prevented the assessment of possible effects of race on the score. Therefore, the scoring system requires validation or adaption in Black and Asian patients, particularly as HCV GT3 is most prevalent in Asia. Additionally, the patient population included in the analysis limits the scoring system to treatment naïve patients. Although the majority of HCV-infected patients worldwide have not yet been treated, treatment-experienced patients and patients with advanced liver disease should be given priority access to DAA-based therapies. RVR was defined as <50 IU/mL in this analysis; more sensitive assays are now in widespread use and it is unclear if the use of a lower threshold to define RVR would produce different results. Although the number of cirrhotic patients was low the scoring system still worked well, with SVR rates in score groups with >10 patients (0–4, 5, and 6) similar to those of patients without cirrhosis. Importantly, fibrosis status was known for all patients in the validation cohort. Host genotype was not included in this analysis because its impact on treatment outcome was not discovered until after the initiation of the PROPHESYS trial [30]. In HCV GT3-infected patients carriers of rs12979860 CC and rs8099917 TT have been shown to be significantly associated with virological response [31–33]. Information on host genotype, if available, could be used in combination with the scoring system and RVR to guide future treatment decisions.

In conclusion, patient selection is known to be important for successful treatment of chronic hepatitis C. The proposed scoring system is easy to calculate and uses baseline characteristics that are readily available in everyday practice to identify treatment-naive Caucasian patients with HCV GT3 infection most likely to respond to treatment with PegIFN alfa-2a/RBV. Application of the scoring system has the potential to identify patients with a high or low probability of achieving an SVR when treated with PegIFN alfa-2a/RBV and will be particularly useful in settings where access to DAAs is restricted.

## Supporting Information

S1 File Prediction formula. (DOCX)

S1 Table Patients’ country of enrollment (development cohort). (DOCX)

S2 Table Patients’ country of enrollment (validation cohort). (DOCX)

S3 Table Summary of logistic regression analysis. (DOCX)

S4 Table Sensitivity, specificity, positive predictive value and negative predictive value of the prediction score for SVR (development cohort). (DOCX)
