# Supplementary material for: A Predictive Model for Selecting Patients with HCV Genotype 3 Chronic Infection with a High Probability of Sustained Virological Response to Peginterferon Alfa-2a/Ribavirin
Source: PLoS One. 2016 Mar 18;11(3):e0150569. doi: 10.1371/journal.pone.0150569 (PMC4798721; doi:10.1371/journal.pone.0150569)
Supplement: S1 File — (DOCX) [file pone.0150569.s001.docx]

# Supporting Information

**Prediction formula**

In addition to using the prediction score in the main text of the paper, the following formula can be used to estimate the probability for SVR in treatment-naive, Caucasian GT3-infected patients.

P (SVR) = 1 / [1 + exp (1.5923 – 1.0108 (if ≤40 yrs) – 0.3494 (if 41 to 55 yrs) – 0.7338 (if <70 kg) – 0.3911 (if 70–<90 kg) – 0.7404 (if noncirrhotic) –0.4859 (if HCV RNA <400,000 IU/mL) – 0.7591 (if platelets >200 x 10^9^/mL ) – 0.5746 (if platelets >100 and ≤200 x 10^9^/mL) – 0.3348 (if ALT ratio ≤2.5).

Examples:

1. A 35-year-old noncirrhotic patient with a bodyweight of 65 kg, HCV RNA level of 350,000 IU/mL, platelet count of 250 x 10^9^/mL and an ALT ratio of 4 (prediction score of 9) has a probability for SVR as follows:

1/[1 + exp (1.5923 – 1.0108 – 0.7338 – 0.7404 – 0.4859 – 0.7591)] = **89.5%**

1. A 50-year-old noncirrhotic patient with a bodyweight of 80 kg, HCV RNA level of 1,650,000 IU/mL, platelet count of 150 x 10^9^/mL and an ALT-ratio of 2 (prediction score of 6) has a probability for SVR as follows:

1/[1 + exp (1.5923 – 0.3494 – 0.3911 – 0.7404 – 0.5746 – 0.3348)] = **69.0%**

1. A 57-year-old patient with cirrhosis, a bodyweight of 95 kg, HCV RNA level of 800,000 IU/mL, platelet count of 80 x10^9^/mL and an ALT-ratio of 3 (prediction score of 0) has a probability for SVR as follows:

1/[(1 + exp (1.5923)] = **16.9%**
