# Supplementary material for: A Predictive Model for Selecting Patients with HCV Genotype 3 Chronic Infection with a High Probability of Sustained Virological Response to Peginterferon Alfa-2a/Ribavirin
Source: PLoS One. 2016 Mar 18;11(3):e0150569. doi: 10.1371/journal.pone.0150569 (PMC4798721; doi:10.1371/journal.pone.0150569)
Supplement: S1 Table — (DOCX) [file pone.0150569.s002.docx]

**S1 Table: Patients’ country of enrollment (development cohort)**

| **Country, n (%)** | **PegIFN alfa-2a + RBV 800 mg; 24 weeks (N=735)** | **PegIFN alfa-2a + RBV 1000/1200 mg; 24 weeks (N=504)** | **Total (N=1239)** |
| --- | --- | --- | --- |
| Albania | 2 (0.3) | 3 (0.6) | 5 (0.4) |
| Algeria | 1 (0.1) | 0 (0.0) | 1 (<0.1) |
| Austria | 60 (8.2) | 6 (1.2) | 66 (5.3) |
| Bahrain | 0 (0.0) | 5 (1.0) | 5 (0.4) |
| Belgium | 81 (11.0) | 45 (8.9) | 126 (10.2) |
| Bosnia-Herzegovina | 7 (1.0) | 2 (0.4) | 9 (0.7) |
| Brazil | 0 (0.0) | 14 (2.8) | 14 (1.1) |
| Canada | 35 (4.8) | 23 (4.6) | 58 (4.7) |
| Croatia | 9 (1.2) | 8 (1.6) | 17 (1.4) |
| France | 105 (14.3) | 80 (15.9) | 185 (14.9) |
| Great Britain | 0 (0.0) | 1 (0.2) | 1 (<0.1) |
| Greece | 67 (9.1) | 13 (2.6) | 80 (6.5) |
| Hungary | 0 (0.0) | 1 (0.2) | 1 (<0.1) |
| Iran | 19 (2.6) | 4 (0.8) | 23 (1.9) |
| Ireland | 17 (2.3) | 2 (0.4) | 19 (1.5) |
| Italy | 127 (17.3) | 144 (28.6) | 271 (21.9) |
| Lebanon | 0 (0.0) | 3 (0.6) | 3 (0.2) |
| Macedonia | 61 (8.3) | 0 (0.0) | 61 (4.9) |
| Poland | 25 (3.4) | 21 (4.2) | 46 (3.7) |
| Portugal | 7 (1.0) | 13 (2.6) | 20 (1.6) |
| Serbia | 58 (7.9) | 13 (2.6) | 71 (5.7) |
| Slovakia | 3 (0.4) | 7 (1.4) | 10 (0.8) |
| Slovenia | 21 (2.9) | 10 (2.0) | 31 (2.5) |
| Sweden | 10 (1.4) | 17 (3.4) | 27 (2.2) |
| United Arab Emirates | 1 (0.1) | 0 (0.0) | 1 (<0.1) |
| USA | 19 (2.6) | 69 (13.7) | 88 (7.1) |
