# Supplementary material for: A Predictive Model for Selecting Patients with HCV Genotype 3 Chronic Infection with a High Probability of Sustained Virological Response to Peginterferon Alfa-2a/Ribavirin
Source: PLoS One. 2016 Mar 18;11(3):e0150569. doi: 10.1371/journal.pone.0150569 (PMC4798721; doi:10.1371/journal.pone.0150569)
Supplement: S2 Table — (DOCX) [file pone.0150569.s003.docx]

**S2 Table: Patients’ country of enrollment (validation cohort)**

| **Country, n ( %)** | **PegIFN alfa-2a + RBV 800 mg; 24 weeks (n=386)** | **PegIFN alfa-2a + RBV 1000/1200 mg; 24 weeks (n=88)** | **Total (N=474)** |
| --- | --- | --- | --- |
| Australia | 38 (9.8) | 9 (10.2) | 47 (9.9) |
| Belgium | 1 (0.3 | 0 (0.0) | 1 (0.2) |
| Brazil | 5 (1.3) | 9 (10.2) | 14 (3.0) |
| Canada | 24 (6.2) | 1 (1.1) | 25 (5.3) |
| Denmark | 0 (0.0) | 2 (2.3) | 2 (0.4) |
| Finland | 4 (1.0) | 3 (3.4) | 7 (1.5) |
| France | 28 (7.3) | 6 (6.8) | 34 (7.2) |
| Germany | 34 (8.8) | 10 (11.4) | 44 (9.3) |
| Great Britain | 0 (0.0) | 1 (1.1) | 1 (0.2) |
| Greece | 4 (1.0) | 2 (2.3) | 6 (1.3) |
| Ireland | 1 (0.3) | 1 (1.1) | 2 (0.4) |
| Italy | 16 (4.1) | 5 (5.7) | 21 (4.4) |
| New Zealand | 9 (2.3) | 3 (3.4) | 12 (2.5) |
| Norway | 1 (0.3) | 4 (4.5) | 5 (1.1) |
| Spain | 36 (9.3) | 10 (11.4) | 46 (9.7) |
| Sweden | 3 (0.8) | 2 (2.3) | 5 (1.1) |
| Netherlands | 2 (0.5) | 1 (1.1) | 3 (0.6) |
| USA | 180 (46.6) | 19 (21.6) | 199 (42.0) |
