# Supplementary material for: A Predictive Model for Selecting Patients with HCV Genotype 3 Chronic Infection with a High Probability of Sustained Virological Response to Peginterferon Alfa-2a/Ribavirin
Source: PLoS One. 2016 Mar 18;11(3):e0150569. doi: 10.1371/journal.pone.0150569 (PMC4798721; doi:10.1371/journal.pone.0150569)
Supplement: S3 Table — (DOCX) [file pone.0150569.s004.docx]

**S3 Table: Summary of logistic regression analysis**

|  | **Univariate Logistic Regression** | | | **Final Multiple Logistic Regression** | | | |
| --- | --- | --- | --- | --- | --- | --- | --- |
| **Baseline characteristic** | **Regression coefficient** | **Odds ratio (95% CI)** | **p-value (effect)^a^** | **Regression Coefficient** | **Odds ratio (95% CI)** | **p-value^b^** | **p-value (effect)^a^** |
| Gender: male vs female | -0.2533 | 0.776 (0.584, 1.031) | 0.0805 |  |  |  |  |
| Planned ribavirin dose (mg/day): 1000/1200 vs. 800 | -0.0271 | 0.973 (0.753, 1.258) | 0.8361 |  |  |  |  |
| Age (yrs): |  |  |  |  |  |  |  |
| ≤40 vs >55 | 1.1778 | 3.247 (2.007, 1.432) | <0.0001 | 1.0108 | 2.748 (1.636, 4.615) | 0.0001 | <0.0001 |
| 40 – ≤55 vs. >55 | 0.3594 | 1.432 (0.897, 2.288) | <0.0001 | 0.3494 | 1.418 (0.862, 2.334) | 0.1619 | <0.0001 |
| Bodyweight (kg) : |  |  |  |  |  |  |  |
| <70 vs ≥90 | 0.7968 | 2.218 (1.544, 3.188) | <0.0001 | 0.7338 | 2.083 (1.406, 3.086) | 0.0003 | 0.0012 |
| 70 – <90 vs ≥90 | 0.4367 | 1.548 (1.111, 2155) | <0.0001 | 0.3911 | 1.479 (1.033, 2.116) | 0.0324 | 0.0012 |
| No cirrhosis vs. cirrhosis / transition to cirrhosis | 1.1293 | 3.094 (2.211, 4.329) | <0.0001 | 0.7404 | 2.097 (1.435, 3.063) | 0.0001 | 0.0001 |
| HCV RNA (IU/mL): |  |  |  |  |  |  |  |
| <400 000 vs. ≥400 000 | 0.5750 | 1.777 (1.345, 2.324) | <0.0001 | 0.4859 | 1.626 (1.205, 2.193) | 0.0015 | 0.0015 |
| Platelets (10^9^/mL): |  |  |  |  |  |  |  |
| >200 vs ≤100 | 1.4928 | 4.450 (2.420, 8.182) | <0.0001 | 0.7591 | 2.136 (1.091, 4.182) | 0.0267 | 0.0680 |
| >100 to 200 vs ≤100 | 1.0081 | 2.740 (1.487, 5.050) | <0.0001 | 0.5746 | 1.776 (0.919, 3.434) | 0.0875 | 0.0680 |
| ALT ratio (xULN): ≤2.5 vs >2.5 | 0.5305 | 1.700 (1.313, 2.200) | <0.0001 | 0.3348 | 1.389 (1.053, 1.854) | 0.0203 | 0.0203 |

^a^ Wald chi-square test for each variable; ^b^ Wald chi-square test for each regression coefficient

**S4 Table: Sensitivity, specificity, positive predictive value and negative predictive value of the prediction score for SVR (development cohort)**

|  | **True** | | **False** | |  |  |  |  |
| --- | --- | --- | --- | --- | --- | --- | --- | --- |
| **Score** | **SVR** | **Non-SVR** | **SVR** | **Non-SVR** | **Sensitivity (%)** | **Specificity (%)** | **PPV (%)** | **NPV (%)** |
| ≥ 1 | 865 | 0 | 301 | 0 | 100 | 0 | 74 | - |
| ≥ 2 | 864 | 3 | 298 | 1 | 100 | 1 | 74 | 75 |
| ≥ 3 | 858 | 8 | 293 | 7 | 98 | 3 | 75 | 53 |
| ≥ 4 | 844 | 32 | 269 | 21 | 98 | 11 | 76 | 60 |
| ≥ 5 | 801 | 77 | 224 | 64 | 93 | 26 | 78 | 55 |
| ≥ 6 | 725 | 124 | 177 | 140 | 84 | 41 | 80 | 47 |
| ≥ 7 | 579 | 181 | 120 | 286 | 67 | 60 | 83 | 39 |
| ≥ 8 | 391 | 242 | 59 | 474 | 45 | 80 | 87 | 34 |
| ≥ 9 | 195 | 278 | 23 | 670 | 23 | 92 | 89 | 29 |
| ≥ 10 | 41 | 296 | 5 | 824 | 5 | 98 | 89 | 26 |

Sensitivity = 100 x true SVR/(true SVR + false non-SVR); Specificity = 100 x true non-SVR/(true non-SVR + false SVR); PPV = 100 x true SVR/(true SVR + false SVR); NPV= 100 x true non-SVR/(true non-SVR + false non-SVR
