# Supplementary material for: A Predictive Model for Selecting Patients with HCV Genotype 3 Chronic Infection with a High Probability of Sustained Virological Response to Peginterferon Alfa-2a/Ribavirin
Source: PLoS One. 2016 Mar 18;11(3):e0150569. doi: 10.1371/journal.pone.0150569 (PMC4798721; doi:10.1371/journal.pone.0150569)
Supplement: S4 Table — (DOCX) [file pone.0150569.s005.docx]

**S4 Table: Sensitivity, specificity, positive predictive value and negative predictive value of the prediction score for SVR (development cohort)**

|  | **True** | | **False** | |  |  |  |  |
| --- | --- | --- | --- | --- | --- | --- | --- | --- |
| **Score** | **SVR** | **Non-SVR** | **SVR** | **Non-SVR** | **Sensitivity (%)** | **Specificity (%)** | **PPV (%)** | **NPV (%)** |
| ≥ 1 | 865 | 0 | 301 | 0 | 100 | 0 | 74 | - |
| ≥ 2 | 864 | 3 | 298 | 1 | 100 | 1 | 74 | 75 |
| ≥ 3 | 858 | 8 | 293 | 7 | 98 | 3 | 75 | 53 |
| ≥ 4 | 844 | 32 | 269 | 21 | 98 | 11 | 76 | 60 |
| ≥ 5 | 801 | 77 | 224 | 64 | 93 | 26 | 78 | 55 |
| ≥ 6 | 725 | 124 | 177 | 140 | 84 | 41 | 80 | 47 |
| ≥ 7 | 579 | 181 | 120 | 286 | 67 | 60 | 83 | 39 |
| ≥ 8 | 391 | 242 | 59 | 474 | 45 | 80 | 87 | 34 |
| ≥ 9 | 195 | 278 | 23 | 670 | 23 | 92 | 89 | 29 |
| ≥ 10 | 41 | 296 | 5 | 824 | 5 | 98 | 89 | 26 |

Sensitivity = 100 x true SVR/(true SVR + false non-SVR); Specificity = 100 x true non-SVR/(true non-SVR + false SVR); PPV = 100 x true SVR/(true SVR + false SVR); NPV= 100 x true non-SVR/(true non-SVR + false non-SVR
